# On the Causes Which Influence the Size of the Pupil

**Published:** 1816-08

**Authors:** Nicholas Litteton

**Affiliations:** of the Royal College of Surgeons.


THE LONDON
### Medical and Physical Journal.

2 OF VOL. XXXVI.] AUGUST, 1816. [NO. 210.

"For many fortunate discoveries in medicine, and for the detection of numerous errors, the world is indebted to the rapid circulation of Monthly "Journals; and there never existed any work to which the Faculty in "EUROPE and AMERICA were under deeper obligations than to the "Medical and Physical Journal of London, now forming a long, but an "invaluable, series."—RUSH.

*For the London Medical and Physical Journal.*

### *On the Causes which influence the Size of the Pupil;* by NICHOLAS LITTETON, Esq. of the Royal College of Surgeons.

THE almost impossibility of knowing every fact and observation which may have been mentioned by authors on medical subjects alone, causes me to doubt the originality of most of the following communications. The utility of many of the facts hereafter mentioned will, I think, be plain to every medical person; and, perhaps, some of the observations or remarks, which at present appear merely curious, may, at some future period, be found useful.

These considerations induce me to submit to public view whatever may be contained in the following pages:—

### *On the Causes which affect the Size of the Pupil.*

As the variable size of the pupil cannot be accounted for on the mechanical principles of elasticity or vascular distension, the muscularity of the iris must be admitted, although its muscular fibres may not be satisfactorily detected. Its muscularity is of a peculiar nature,—the *vita propria* of Blumenbach. But, to those who consider the power of contraction and relaxation a sufficient proof of muscularity, it is, perhaps, not necessary to announce discoveries of new words, or of such microscopic or ideal distinctions of animal powers, as the words contractility, muscularity, *vita propria*, &c. seem to convey. If we would have other proofs before we admit of the existence of muscularity in any organ, we should, perhaps, be compelled to deny the muscularity of many parts of the body now universally allowed to be possessed of that power.

The adjectives voluntary and involuntary express the muscular peculiarities of some parts. But, if we examine the laws which govern, and the causes which affect, the different ferent muscular parts, we shall find these peculiarities to be more extensively varied. The muscles which move the limbs and external parts, the sphincters, the diaphragm, the heart, the alimentary canal, the urinary and gall bladders, the ducts and vessels, the muscles of hearing and vision, the uterus and fallopian tubes, have all some peculiarity, which, in each, is admirably adapted to its particular use.

Analogous to these variations in muscularity, are the peculiarities of sensibility of different nervous expansions; one being for hearing, another for seeing, smelling, &c.

The peculiarity of contraction in the iris is detected by attending to its state when influenced by different causes. The effects of such causes must be either to produce diminution or enlargement of the pupil. The subject may be therefore described with propriety under two heads: first, such causes as lessen the size of the pupil; and, secondly, such as tend to, or actually enlarge its area.

Before proceeding further, it seems necessary to describe the appearance of the iris more minutely than has been heretofore done. It is well known to vary in colour in different persons. The irides of the same person I have also seen to differ, one being of a light color, whilst the other was very dark. And even the iris itself will sometimes differ in color, one part of its annulus included between two radii being of one color, and the other part of a quite different color.

There may be always remarked also a distinct difference of color between the external part of the iris (the peripheral annulus), which is attached to the corpus ciliare and its internal part (the central annulus).

Wherever we observe the iris, it must necessarily be exposed to one of the causes which diminishes the size of the pupil, viz. light. In certain states of the body, however, this exposure has no effect on it. In health, during our waking hours, the pupil will be found of a greater or less diameter inversely as the degree of light to which it is exposed; and it will be observed to instantaneously vary with every sudden increase or diminution of its influence. In the least degrees of light in which it is possible to see the iris, the area of the pupil will be found increased proportionally as the central annulus decreases, until (except in the aged) even the whole of this annulus disappears. In the greatest degrees of light, the pupil will be found decreased to between one-third and one-fourth of the diameter of the central ring, and in aged subjects the pupil may be seen of a much smaller size. I found the pupils no larger than a common pin's head in a woman, æt. 75, who had been in a comatose state for two or three weeks about four or five years before.

The

The causes which enlarge or diminish the size of the pupil affected her pupils in a very trifling degree. This state of the pupil is found to prevail in all other aged subjects, but I have never remarked it in such an extreme degree in any other instance.

The quickness and great extent to which the iris contracts, does not, perhaps, exist in other muscular fibres; when we also recollect that this contraction seems chiefly in the central annulus, the great difference in this respect from other known muscular contractions is extreme. The voluntary muscles do not contract more than from one-tenth to one-fifth of their whole length. And, although some involuntary muscular parts, as the uterus and vesica urinaria, contract to an extreme degree, yet these differ from the iris in their contractile velocity.

Another cause which diminishes the size of the pupil is the exertion of the eye, whilst viewing distant objects. In order to observe the lessened size of the pupil from this cause, it is best, or even necessary, that the eyes should not be exposed to much light; either because the power of contraction in the iris is inversely as the degree of light, or, I conceive, if more light is present than is necessary to cause the extreme of diminution in the size of the pupil, that the light which is more than is necessary to cause effect may counteract the disposition to enlargement in the pupil when near objects are viewed. It is easily observed in that degree of light which prevails soon after the setting, or before the rising, of the sun.

The telescopic power of the eye depends only in a trifling degree on this cause of diminished pupil, for the following reasons:—When looking with both eyes at a far distant object in a moderate degree of light, the pupils will be lessened in size, and if, whilst looking at the same object, one of the eyes is suddenly closed, the pupil of the other will increase in size,* and still the distant object will be as distinctly seen as before. There exists, therefore, a cause of enlarged pupil in all healthy eyes, which is equal to the counteraction of this cause of diminished pupil, without at all affecting the telescopic power.

The pupil may be fixedly enlarged, or fixedly diminished in size; this may be from a diminution or loss of power in the radiated fibres, or an increased power in the circular fibres of the iris; that is, from an increased power in the radiated, or loss of it in the circular, fibres. When the pu-

* This is from sympathy with the enlarged pupil of the closed eye, which will be noticed hereafter.

pil is fixedly enlarged in size, light causes a feeling of fatigue until the eye becomes accustomed to the change, after which, near or distant objects are again viewed with as great accuracy as before. This, I had an opportunity of remarking in a man, æt. 50, who had had some injury in one of his eyes many years before, which, having destroyed the power of contraction in, or perhaps even the muscular substance of three-fourths of the central annulus, caused it always to remain fixedly enlarged, yet its telescopic power was as complete as in the other eye, which was perfect. The only inconvenience he experienced after recovering from the injury was, an inability of bearing any sudden increase of light, as well as before, which, however, after a time, also ceased. In the fixed state of the pupil, caused by belladonna, I could see distinctly common letter-print at a distance of two inches in obscure day-light with the unaffected eye, whereas, the same print became hazy to the affected eye at four inches. Consistent with the opinion that this power depends on the state of the pupil, objects ought to have been seen nearer with the affected eye than with the other. This fact, with respect to the telescopic power, some may think a sufficient proof, that the belladonna, whilst it affects the iris, does also, in some degree, affect the retina.

The great probability that the retina is affected in those cases of fixed pupil, where paralytic symptoms are or have been present, prevents the drawing any conclusions from such sources. I shall, however, give two cases: one in a woman, æt. 56; she had, for several weeks, felt numbness in the left arm, with sensation of fulness in the head and giddiness, mistiness of vision, and aching of the eye-balls. The state of the left pupil is such as is commonly observed in persons of the same age. The right pupil in a moderate degree of light is not more than half the size of the left, but it still contracts by sympathy or increase of light. She can see the print of a duodecimo prayer-book with both eyes, best at the distance of fifteen inches; at six or eight inches, the letters become hazy, as they also do when more distant than three feet. The distance between six inches and three feet may here with propriety be called the range of vision for that size of object. With the left eye alone, she thinks she sees better than with both, the range of vision being still more extensive. The right eye has a small range of vision, the same letters being hazy when nearer than ten or twelve inches, and also when removed beyond fifteen or sixteen inches.

The other case is in a woman, æt. 65. The pupil of her left eye has been fixedly enlarged nearly to the whole extent of the central annulus for twelve years. This enlarged state, together with strabismus, was consequent on an attack of violent pain in the left and anterior part of the head. She cannot read common print with the affected eye since, but the distance of most perfect vision is nearer than in the sound eye, with which she can read common print. The range of vision is very much diminished in the diseased eye.

He, therefore, who attempts to prove the possibility of a perfect image on the retina in all cases of distinct vision, resembles the hypochondriac who fancies himself glass. They may reason correctly, they may not overlook any optical facts or mathematical deductions, but the fancy of the one is fallacious, and the attempt of the other vain. A range of vision, of many feet, of many miles, to be effected by an instrument which may be included in a nutshell, must defy all human opticians; and can never be effected, but through the help of a retina capable of receiving impressions distinctly, although the image is imperfect. Supposing, therefore, all the trifling causes of telescopic power which have been alleged were removed, I ask, whether this grand cause would not easily accommodate itself to their absence, and whether the range of vision would not soon be as perfect without them?

The influence of volition on the contraction of the iris in the human being has been mentioned, but it seems so improbable and so imperfectly proved, that it ought not to be considered a cause of diminished pupil until more instances are observed.

The voluntary command of the heart appears to me in the same light, and Dr. Cheyne's case may be, perhaps, otherwise accounted for. Many there are, no doubt, who can indirectly check the pulsation of the arteries in their upper extremities, and, at the same time, prevent the beating of their hearts from being felt; perhaps, also a short suspension of expiration when the lungs are distended with air, might cause syncope in one so diseased as Colonel Townsend.

The iris of the parrot, however, seems to be influenced by volition.

One cause of diminished pupil, which, I believe, has been hitherto unknown, is sleep. During this state of the body, the pupil will be found at, perhaps, its extreme of diminution, especially when the sleep is profound. I have remarked this fact at various ages, and in different diseases, and have no doubt that it is always the case in all instances, where light is capable of affecting the pupil during the waking state.

An

An attention to this fact will enable any one to detect feigned sleep.

A knowledge which is of, perhaps, equal importance is, that this same state of the pupil prevails in coma and stupor from various causes. I have remarked it in asphyxia from carbonic acid gas; during the sleep or stupor from opium; also, in the stupor which occurs in some infantile diseases. And, during the insensible state which occurs in concussion immediately after the blow, as well as in the coma which succeeds, the pupils are in the same state.

These causes gradually tend, in proportion to their prevalence, to lessen the extreme of enlargement of the pupil during the waking state. Hence the diminished area of the enlarged pupil in old age, and its very small size in the case before-mentioned, may be accounted for. I am fully aware of the still imperfect state of our knowledge on the present subject. Numerous questions are suggested to my mind even whilst I am writing, which I hope hereafter to be enabled to answer.

The second part of this subject, viz. the causes which enlarge the area of the pupil, I shall communicate at a future period, when I hope additional facts will tend to the elucidation of various anomalous appearances of the pupil.

*Padstow; June* 10, 1816.

In a former communication, in vol. xxxiv. page 454, line fifth, for *conclusion* read *non-existence.*

---

We have found so many difficulties in procuring satisfactory engravings, that it was at length determined to print the present communication, and preserve the illustrations till Mr. Litteton favours us with the rest of his remarks.—Edit.

---

*For the London Medical and Physical Journal.*

